# A Bayesian approach for detecting a disease that is not being modeled

**DOI:** 10.1371/journal.pone.0229658

**Published:** 2020-02-28

**Authors:** John M. Aronis, Jeffrey P. Ferraro, Per H. Gesteland, Fuchiang Tsui, Ye Ye, Michael M. Wagner, Gregory F. Cooper

**Affiliations:** 1 Real-time Outbreak and Disease Surveillance (RODS) Laboratory, Department of Biomedical Informatics, University of Pittsburgh, Pittsburgh, Pennsylvania, United States of America; 2 Department of Biomedical Informatics, University of Utah, Salt Lake City, Utah, United States of America; International Prevention Research Institute, FRANCE

## Abstract

Over the past decade, outbreaks of new or reemergent viruses such as severe acute respiratory syndrome (SARS) virus, Middle East respiratory syndrome (MERS) virus, and Zika have claimed thousands of lives and cost governments and healthcare systems billions of dollars. Because the appearance of new or transformed diseases is likely to continue, the detection and characterization of emergent diseases is an important problem. We describe a Bayesian statistical model that can detect and characterize previously unknown and unmodeled diseases from patient-care reports and evaluate its performance on historical data.

## Introduction

Over the past decade, outbreaks of new or reemergent viruses such as severe acute respiratory syndrome (SARS) virus, Middle East respiratory syndrome (MERS) virus, and Zika have claimed thousands of lives and cost governments and healthcare systems billions of dollars. Whether these outbreaks were caused by increased international travel, mutating viruses, or climate change, it is clear that our generation and future generations must find ways to recognize and contain outbreaks of new viruses quickly.

The ultimate goal should be the *prediction* of emergent diseases before they strike the human population [1]. This is a lofty goal that will require dramatic advances in pathology, genetics, and ecology, along with major advances in computational science and public health practice. A complementary and achievable near-term strategy is *surveillance* of human populations for early signs of infectious outbreaks [2]. While still a formidable task, we can build on comprehensive surveillance systems already in place in the United States and much of the world [3–7].

The simplest *univariate* detection algorithms [8] track a time-series of a single value, such as emergency department visits or thermometer sales, and look for significant deviations from a baseline level of expected activity. *Multivariate* systems [9] combine several indicators into a single compound indicator in seeking to increase performance. However, these systems suffer from two problems. First, if an outbreak of a new disease occurs during a larger outbreak of a known disease, it might not be noticed. For instance, imagine if a new disease causes fever. People with this disease might purchase thermometers. However, if an outbreak of this new disease occurs during a large outbreak of influenza the increased thermometer sales due to the new disease would be overshadowed by the number of thermometer sales due to influenza. Second, they assume that *the future will be like the past*, and that outbreaks of influenza and other known diseases occur at the same time each year. For instance, suppose that the system expects increased thermometer sales during the month of January because that is when outbreaks of influenza typically occur but, in fact, there is no January outbreak of influenza in the current year. Then, an increase in thermometer sales in January due to an outbreak of a new disease might well be attributed to the expected outbreak of influenza and dismissed.

The WSARE (*What’s Strange About Recent Events*) system [10] addresses these issues by representing the joint distribution of patient data with a Bayesian network that includes several *environmental attributes* to represent *influenza activity*, *season*, *weather*, etc. and several *response attributes* to represent patient attributes such as *age*, *gender*, *location*, and *reported symptom* (which is similar to a patient’s chief complaint and takes a value from *none*, *respiratory problems*, *nausea*, or *rash*). The network is conditioned on the current values of the enviromental attributes to create a conditional joint distribution of response variables for the current day. Thus, the conditional joint distribution represents what would be expected for the current day if there are no outbreaks of new diseases. Thus, WSARE does not take a strictly technical approach to predicting cyclic patterns, but rather incorporates current conditions. The WSARE system then searches for rules that describe significant differences between the actual current data and the conditional joint distribution. For instance, if there are many patients with fever in the current data, but the conditional joint distribution predicted few patients with fever, WSARE would report this. The WSARE system has two important shortcomings, however. First, as with time-series methods, if an outbreak of a new disease occurs during a large outbreak of influenza it might not be noticed. Second, it can be misled by outbreaks of other known diseases such as RSV, parainfluenza, hMPV, etc. For instance, if the environmental attribute *influenza activity* is low, but there is an outbreak of RSV, the resulting surge of patients with respiratory ailments would cause a false alarm. (We note that these shortcomings could be corrected by adding additional enviromental attributes for each known disease and additional response attributes for a set of clinical findings sufficient to distinguish between different respiratory illnesses).

A patient’s chief complaint is a concise statement of the symptom or problem that caused the patient to seek medical care. Often stated in the patient’s own words, a chief complaint typically does not mention predefined syndromes or disease categories. Keyword-based systems can operate in real-time to identify anomalies or clusters of unusual findings from text [11]. [12] describes a semantic scan sytem that infers a set of topics (probability distributions over words) from the free-text of chief complaints. The system learns one set of topics using past data and a second set of topics from the most recent 3-hour moving window, assigns each patient case to its most likely topic, then looks for anomalous counts and clusters of topics. [13] extends this approach to identify clusters also based on geography or social patterns.

A distinctive feature of an infectious outbreak is an initial period of exponential growth. [14] describes a Bayesian system, based on moving windows of ILI records, to detect a period of exponential growth that can signal the start of an outbreak. That system uses Bayes’ factors to determine when it is likely that an outbreak has started.

This paper describes a Bayesian modeling approach called DUDE (*Detection of Unmodeled Diseases from Evidence*) that can recognize outbreaks of new forms of *influenza-like illness* (ILI) and create clinical characterizations of them. We demonstrate its operation on data from real-world outbreaks including an outbreak of Enterovirus EV-D68. DUDE avoids the shortcomings of previous sytems by building probabilistic models of normal (baseline) ILI activity using a large set of patient findings extracted from patient-care reports using natural language processing. (This approach to ILI detection and characterization was developed in [15]). It then looks for statistically significant deviations from baseline normal activity. Thus, DUDE does not rely on just a small set of findings (as might be extracted from patients’ chief complaints). Also, it does not model temporal patterns and therefore does not assume that the present is like the past. Finally, by removing cases of known forms of ILI (such as influenza, RSV, etc.) from the input data, it can recognize new, emergent kinds of ILI.

## Materials and methods

### Software design

DUDE uses emergency department (ED) reports of patients with an ILI (defined as patients with fever and cough or sore throat) who have definitively tested negative for a set of common viruses. Each report has been processed using natural language processing (NLP) to extract binary features for a set of symptoms relevant to the diagnosis of ILI.

We create two *windows* on the data. The *baseline window* includes patient records from a controlled time period when no outbreaks of new viruses are suspected. DUDE collects statistics about rates of symptoms among patients in the baseline window to model *background ILI*. The *monitor window* includes patient records from a later time period. DUDE then computes the likelihood of the data in the monitor period assuming that only background ILI is present, and the likelihood of the data in the monitor period assuming that both background ILI and a different *unmodeled* ILI is present. We then compute the odds that an unmodeled ILI is present. Note that our data are collected in hospitals. The number of patients with a disease who go to a hospital is certainly related to the number of cases in the general population. Even if this is a simple linear relationship it is hidden since we have no way to directly count the number of people in the general population with a disease We avoid the difficulty of determining this relationship by working solely with ED data.

**Fig 1:**
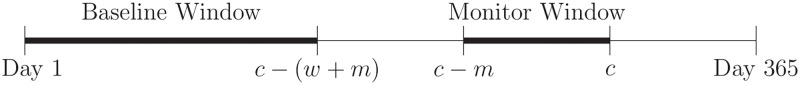
Baseline and monitor windows.

The dataset includes patient records from one outbreak year (from June 1 of one year through May 31 of the next year), and parameters including the length of a wait period *w*, and the length of a monitor window *m*. For each current day *c*, the baseline window includes data from days 1 through *c* − (*w* + *m*) and the monitor window includes data from days *c* − *m* through *c*. For the experiments reported in this paper we set *w* = 14, *m* = 28, and start looking for outbreaks on September 1 (day *c* = 93). Starting with *c* = 93 ensures that the baseline window includes data from at least 50 days which is sufficient to characterize baseline ILI. See Fig 1.

DUDE was implemented in Java running under Ubuntu Linux. All experiments were run on a single 2.5 GHz processor.

### Mathematical modeling

The baseline and monitor windows contain patients who have definitively tested negative for a set of known types of ILI. Therefore, both the baseline and monitor windows contain patients who each have either *background* ILI, denoted B, or an unmodeled ILI, denoted U. We assume that B and U are disjoint and that few (if any) patients with U appear in the baseline window.

Let #B and #U be the number of patients in the monitor window with B or U respectively. We do not know the diagnosis of any patient, the values of #B or #U, or even if #B=0 or #U=0. In fact, our objective is to determine if #U>0.

Each patient *p* has a set of boolean-valued findings $\bar{f}\left(p\right)=\left\{f_{1}\left(p\right),\ldots ,f_{n}\left(p\right)\right\}$ (where *f*_*i*_(*p*) is true if patient *p* has finding *i*). We make two independence assumptions:

$$\begin{array}{c} P\left(\bar{f}\left(p_{1}\right),\ldots ,\bar{f}\left(p_{N}\right)|disease\left(p_{1}\right),\ldots ,disease\left(p_{N}\right)\right)=\prod_{i=1}^{N}P\left(\bar{f}\left(p_{i}\right)|disease\left(p_{i}\right)\right) \end{array}$$ (1)

$$\begin{array}{c} P\left(\bar{f}\left(p\right)|disease\left(p\right)\right)=\prod_{i=1}^{n}P\left(f_{i}\left(p\right)|disease\left(p\right)\right) \end{array}$$ (2)

where N=#B+#U is the total number of patients in the monitor window and *disease*(*p*) is the disease of patient *p* (either B or U). The first assumption says that each patient’s findings depend only on his or her disease. The second says that a patient’s findings are independent given their disease.

For each finding *f*, let:

$$\begin{array}{c} \theta_{B,f}=P\left(f\left(p\right)=T|disease\left(p\right)=B\right) \end{array}$$ (3)

$$\begin{array}{c} \theta_{U,f}=P\left(f\left(p\right)=T|disease\left(p\right)=U\right) \end{array}$$ (4)

That is, $\theta_{B,f}$ is the probability that a patient with disease B has finding *f*, and $\theta_{U,f}$ is the probability that a patient with disease U has finding *f*. We estimate each $\theta_{B,f}$ with:

$$\begin{array}{c} \theta_{B,f}=\left(\#f_{b}+1\right)/\left(\#total_{b}+2\right) \end{array}$$ (5)

where #*f*_*b*_ is the number of patients in the baseline window with finding *f* and #*total*_*b*_ is the total number of patients in the baseline window. This estimate is based on our assumption that the baseline window includes only patients with background ILI. Also, let #*f*_*m*_ be the number of patients in the monitor window with finding *f*, and let $\left\{\theta_{B,f}\right\}$, $\left\{\theta_{U,f}\right\}$, and {#*f*_*m*_} denote the sets of these values where *f* ranges over the set of findings.

Given that #B and #U patients in the monitor window have diseases B and U, respectively, the probabilities $\theta_{B,f}$ and $\theta_{U,f}$ from Eqs 3 and 4, and Assumptions 1 and 2, the probability that exactly *i* of the patients with B and *j* of the patients with U have finding *f* is:

$$\begin{array}{c} bin\left(\#B,i,\theta_{B,f}\right)\;bin\left(\#U,j,\theta_{U,f}\right) \end{array}$$ (6)

where *bin* is the binomial distribution. (That is, *bin*(*n*, *r*, *p*) is the probability of choosing *r* items from a set of *n* items if each is selected independently with probability *p* if 0 ≤ *r* ≤ *n*, and 0 otherwise).

Since #*f*_*m*_ patients in the monitor window have finding *f*, then between 0 and #*f*_*m*_ of those patients have disease B and the remainder must have disease U. Since these are disjoint we have:

$$\begin{array}{c} P\left(\#f_{m}|\#B,\#U,\theta_{B,f},\theta_{U,f}\right)=\sum_{i=0}^{\#f_{m}}\left[bin\left(\#B,i,\theta_{B,f}\right)\;bin\left(\#U,\#f_{m}-i,\theta_{U,f}\right)\right] \end{array}$$ (7)

This is simply the sum of probabilities over all possible ways that #*f*_*m*_ patients with finding *f* can be divided between a set of #B patients and a set of #U patients. We are ignorant of the values of $\theta_{U,f}$ so we assume a uniform PDF for it and integrate over [0, 1]:

$$\begin{array}{ccc} P(\#f_{m}|\#B,\#U,\theta_{B,f}) & = & \int_{0}^{1}\sum_{i=0}^{\#f_{m}}\left[bin\left(\#B,i,\theta_{B,f}\right)\;bin\left(\#U,\#f_{m}-i,\theta_{U,f}\right)\right]\;d\theta_{U,f} \end{array}$$ (8)

$$\begin{array}{ccc} & = & \frac{1}{\#U+1}\sum_{i=0}^{\#f_{m}}bin\left(\#B,i,\theta_{B,f}\right) \end{array}$$ (9)

The details of this derivation are in S1 Appendix.

The evidence consists of {#*f*_*m*_}, which is the set of counts of each finding in the monitor window. Then:

$$\begin{array}{c} P\left(\left\{\#f_{m}\right\}|\#B,\#U,\left\{\theta_{B,f}\right\}\right)=\prod_{f\in findings}P\left(\#f_{m}|\#B,\#U,\theta_{B,f}\right) \end{array}$$ (10)

The details of this derivation are also in S1 Appendix. Eq 10 allows us to compute the probability of the evidence in the monitor window, {#*f*_*m*_}, given values for #B, #U and each $\theta_{B,f}$. We do not know #B or #U specifically, but we do know that #B+#U=N where *N* is the total number of patients in the monitor window, so:

$$\begin{array}{c} P\left(\left\{\#f_{m}\right\}|\#U,\left\{\theta_{B,f}\right\}\right)=P\left(\left\{\#f_{m}\right\}|\#B=N-\#U,\#U,\left\{\theta_{B,f}\right\}\right) \end{array}$$ (11)

The probability of the evidence {#*f*_*m*_} given that at least one patient in the monitor window has the unmodeled disease U is:

$$\begin{array}{c} P\left(\left\{\#f_{m}\right\}|\#U>0,\left\{\theta_{B,f}\right\}\right)=\frac{1}{N}\sum_{\#U=1}^{N}P\left(\left\{\#f_{m}\right\}|\#B=N-\#U,\#U,\left\{\theta_{B,f}\right\}\right) \end{array}$$ (12)

assuming that it is equally likely that 1 through *N* patients have U. Also, the probability of the evidence given that no patient in the monitor window has the unmodeled disease is:

$$\begin{array}{c} P\left(\left\{\#f_{m}\right\}|\#U=0,\left\{\theta_{B,f}\right\}\right)=\prod_{f\in findings}bin\left(N,\#f_{m},\theta_{B,f}\right) \end{array}$$ (13)

Putting together Eqs 12 and 13, we find the odds that some patients in the monitor window have an unmodeled disease:

$$\begin{array}{c} \frac{P(\left\{\#f_{m}\right\}|\#U>0,\left\{\theta_{B,f}\right\})}{P(\left\{\#f_{m}\right\}|\#U=0,\left\{\theta_{B,f}\right\})} \end{array}$$ (14)

Note that Eq 14 is a *Bayes factor* and can be used to evaluate the weight of the data in favor of the presence of an unmodeled disease.

### Data

The data consists of patient-care reports from emergency departments in the Intermountain Healthcare system in Salt Lake County, Utah from June 1, 2010 through May 31, 2015. These emergency departments capture about 55% of emergency department visits in Salt Lake County. Each report is processed with natural language processing software [16] to extract a set of 65 medical findings that clinicians determined are relevant to the diagnosis of influenza-like illnesses (listed in Table 1). The data were joined to a database of results of laboratory tests. There were a total of 944, 562 patient records. After a strict syntax check of records 3, 063 were removed due to inappropriate laboratory codes leaving 941, 499 reports. From these, we selected 32, 249 reports of patients who definitively tested positive for exactly one of influenza, respiratory syncytial virus (RSV), parainfluenza, or human metapneumovirus (hMPV), or were negative for all four tested diseases.

**Table 1:** Medical findings that clinicians determined are relevant to the diagnosis of influenza-like illnesses.

abdominal pain	dyspnea	productive cough
abdominal tenderness	grunting	rales
abnormal chest radiograph	headache	reported fever
acute onset	hemoptysis	respiratory distress
age under six years	hoarseness	rhonchi
anorexia	hypoxemia	rigor
apnea	ill appearing	runny nose
arthralgia	infiltrate	seizure
barking cough	influenza like illness	sore throat
bilateral acute conjunctivitis	malaise	staccato cough
bronchiolitis	myalgia	streptococcal pharyngitis
bronchitis	nasal flaring	stridor
cervical lymphadenopathy	nausea	stuffy nose
chest pain	nonproductive cough,	tachypnea
chest wall retractions	other abnormal breath sounds	toxic appearance
chills	other cough	uri
conjunctivitis	other pneumonia	viral pneumonia
crackles	paroxysmal cough	viral syndrome
croup	pharyngitis diagnosis	vomiting
cyanosis	pharyngitis on exam	weakness or fatigue
decreased activity	poor feeding	wheezing
diarrhea	poor response to antipyretics	

**Fig 2:**
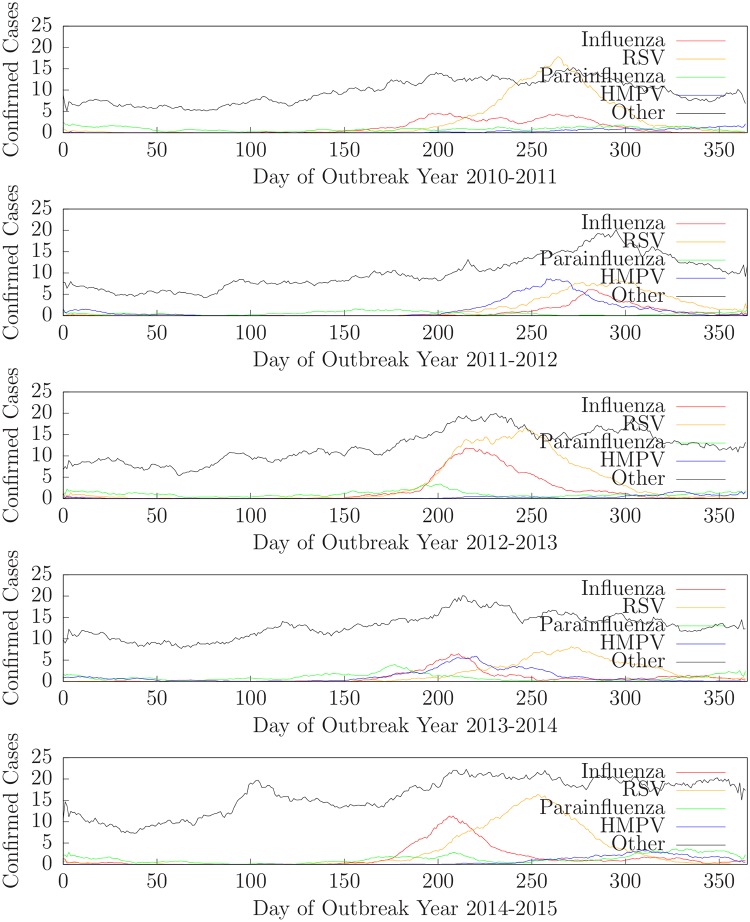
Daily counts of confirmed cases of influenza, RSV, parainfluenza, hMPV, and other for outbreak years 2010-2011 through 2014-2015.

The data is divided into five years: *outbreak year 2010-2011* contains data from June 1, 2010 through May 31, 2011, *outbreak year 2011-2012* contains data from June 1, 2011 through May 31, 2012, etc. For each outbreak year, we designate June 1 to be *day 1*. Fig 2 shows the daily counts (14-day moving averages) of confirmed cases of influenza, RSV, parainfluenza, hMPV, and other (*e.g.* negative for all four) for Intermountain Healthcare emergency departments in Salt Lake County for outbreak years 2010-2011 (top) through 2014-2015 (bottom).

To search for an unmodeled disease we selected records of patients who were negative for all four tested diseases. We used these records to search for outbreaks of ILI other than influenza, RSV, parainfluenza, or hMPV. *That is, outbreaks of unknown or unmodeled diseases.* There were 3, 487 such records in outbreak year 2010-2011, 3, 666 in outbreak year 2011-2012, 4, 501 in outbreak year 2012-2013, 4, 752 in outbreak year 2013-2014, and 5, 884 in outbreak year 2014-2015. (These were all the cases for which laboratory testing was done and all four of influenza, RSV, parainfluenza, and hMPV were negative.) Thus, we had five outbreak years of data in which to search for outbreaks of unmodeled diseases.

To further test DUDE, we *pretended* to be ignorant of each disease (influenza, RSV, parainfluenza, and hMPV) for each year. That is, for each disease and each year we change the positive tests for that disease to negative and create a new dataset that includes a disease that is ‘unmodeled’ to the sytem but known to us. Thus, we created twenty datasets (four diseases times five years) for experimental purposes that include a well-understood disease that is virtually unmodeled.

### Ethics statement

The research protocol was approved by both institutional IRBs (University of Pittsburgh PRO08030129 and Intermountain Healthcare 1024664). All the research patient data were de-identified.

We cannot directly share the data used in this study because it contains potentially identifying information about individual patients. This is an ethical and legal restriction that has been imposed by Intermountain Healthcare. Interested parties may request access to the data from the Intermountain Healthcare IRB, 8th Avenue & C Street, Salt Lake City, UT 84143, phone (801) 408-1991, email IRB@imail.org.

## Results

### Detecting an unmodeled ILI

We ran the algorithm on each outbreak year using only patients who tested negative for all four of influenza, RSV, parainfluenza, and hMPV. Because they were tested, we assume they have an ILI. However, since all of their tests were negative, their diagnoses were indeterminate. That is, they have some kind of ILI but do not have any of the modeled diseases.

**Fig 3:**
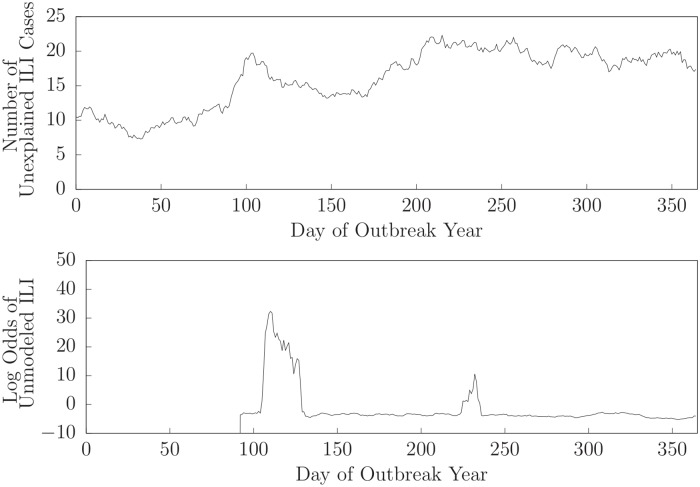
Number of unexplained ILI cases and log-odds that an unmodeled ILI is present for outbreak year 2014-2015.

Fig 3 shows the (logarithm of the) daily odds of the presence of an unmodeled disease in the monitor window for outbreak year 2014-2015. DUDE begins computing odds on day 93 (September 1). The odds of the presence of an unmodeled disease slowly increased and was greater than 1 on day 106 (September 14, 2014) indicating that it was more likely than not that an unmodeled disease was present. After day 106 the odds of the presence of an unmodeled disease increased dramatically. An examination of records in the monitor window at that time showed a prevalence of patients with wheezing, chest wall retractions, runny nose, respiratory distress, crackles, tachypnea, abnormal breath sounds, headache, stuffy nose, and dyspnea. (These are the findings that were at least 25% more likely to occur in a patient in the monitor window than one in the baseline window and were present in at least 10% of the patients in the monitor window).

During this time period, the CDC identified an outbreak of *Enterovirus D68* (EV-D68) [17]. In mid-August 2014 hospitals in Missouri and Illinois notified the CDC of an increase in admissions of children with severe respiratory illness. By September 8, 2014 officials at Primary Children’s hospital in Salt Lake City, Utah suspected the presence of EV-D68 [18], and by September 23, 2014 the CDC confirmed the existence of EV-D68 in Utah [19]. Since August 2014, the CDC and states began doing more testing for EV-D68, and have found that EV-D68 was causing severe respiratory illness in almost all states [20]. Symptoms of EV-D68 include wheezing, difficulty breathing, runny nose, sneezing, cough, body aches, and muscle aches. (Severe symptoms of EV-D68 may also include acute flaccid paralysis [21], but this is not among the symptoms used by DUDE).

**Fig 4:**
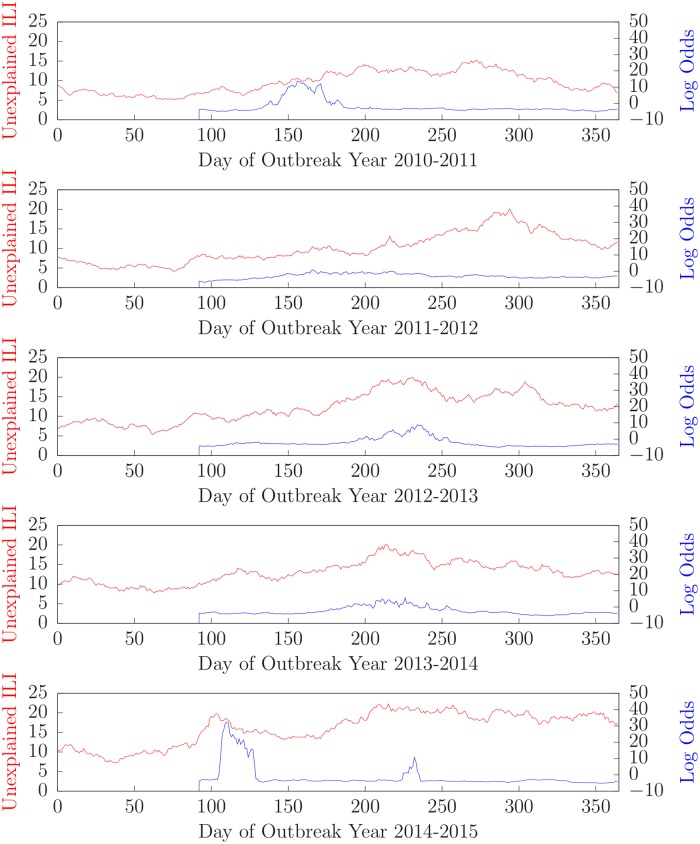
Number of unexplained ILI cases and log-odds that an unmodeled ILI is present for outbreak years 2010-2011 through 2014-2015.

Fig 4 shows the results of running the algorithm on patients who tested negative for all four of influenza, RSV, parainfluenza, and hMPV patients for outbreak years 2010-2011 (top) through 2014-2015 (bottom).

### Accuracy

An outbreak detection system must detect outbreaks in a timely fashion with a minimum of false alarms. Clearly, there is a tradeoff between these two requirements. We can balance these requirements by setting an *alarm threshold*. Each day the likelihood of the presence of an unmodeled disease is greater than this threshold an *alarm* is generated. Ideally, the first alarm is generated on the first day of an outbreak (and each subsequent day while the outbreak is ongoing). Given data for an outbreak year that contains an outbreak of an unmodeled disease, and a specific value for the alarm threshold, the *number of false alarms* is the number of alarms before the start of the outbreak. The *number of days to detection* is the number of days from the start of the outbreak to the first alarm. If the first (false) alarm is before the start of the outbreak, we define the number of days to detection to be zero.

An *activity monitoring operator characteristic* (AMOC) curve is a graph that characterizes the timeliness of a detector [4]. It plots the expected time to detection as a function of the false-positive rate. AMOC curves can be used to compare the timeliness of different detectors. We can illustrate the performance of DUDE with an AMOC curve that plots the number of days to detection versus the number of false alarms for various threshold values. However, to generate an AMOC curve we need to define the start of an outbreak. It is also helpful to average over several outbreaks to measure performance under various circumstances. Since this is not practical to do with unmodeled outbreaks, we used data for outbreaks of known diseases and *pretended* to be ignorant of them, thus making them unmodeled.

There were outbreaks of influenza, RSV, and hMPV in each of the five outbreak years of data. Thus, we had fifteen outbreaks of known diseases. For each disease *D* and each year, we created a dataset that included the records of patients who tested negative for all of the tested diseases in that year, plus the records of patients who tested positive for disease *D* in that year. *Again, we pretended to be ignorant of disease D and call it an assumed unmodeled disease.* To calculate the start date of a modeled outbreak we measure the baseline frequency of positive laboratory tests from June 1 (the first day of data) through September 30 (the last day that an outbreak is unlikely to occur). Then for dates after September 30 we signal the start of an outbreak when the frequency increases significantly for several consequtive days. Specifically:

1. Set the baseline *b* = 122, window size *w* = 7, notify period *n* = 7, and threshold *t* = 0.1.
2. Find the average number of lab-confirmed cases for each window in the baseline period from day 1 (June 1) through day *b* (September 30 = 122). This defines a Poisson distribution.
3. For each window ending on some day after *b* count the number of lab-confirmed cases in that window and find its probability.
4. If the probability of the number of cases is below the threshold for *n* consequtive windows claim that an outbreak started on the first day of the first of the *n* windows.

**Fig 5:**
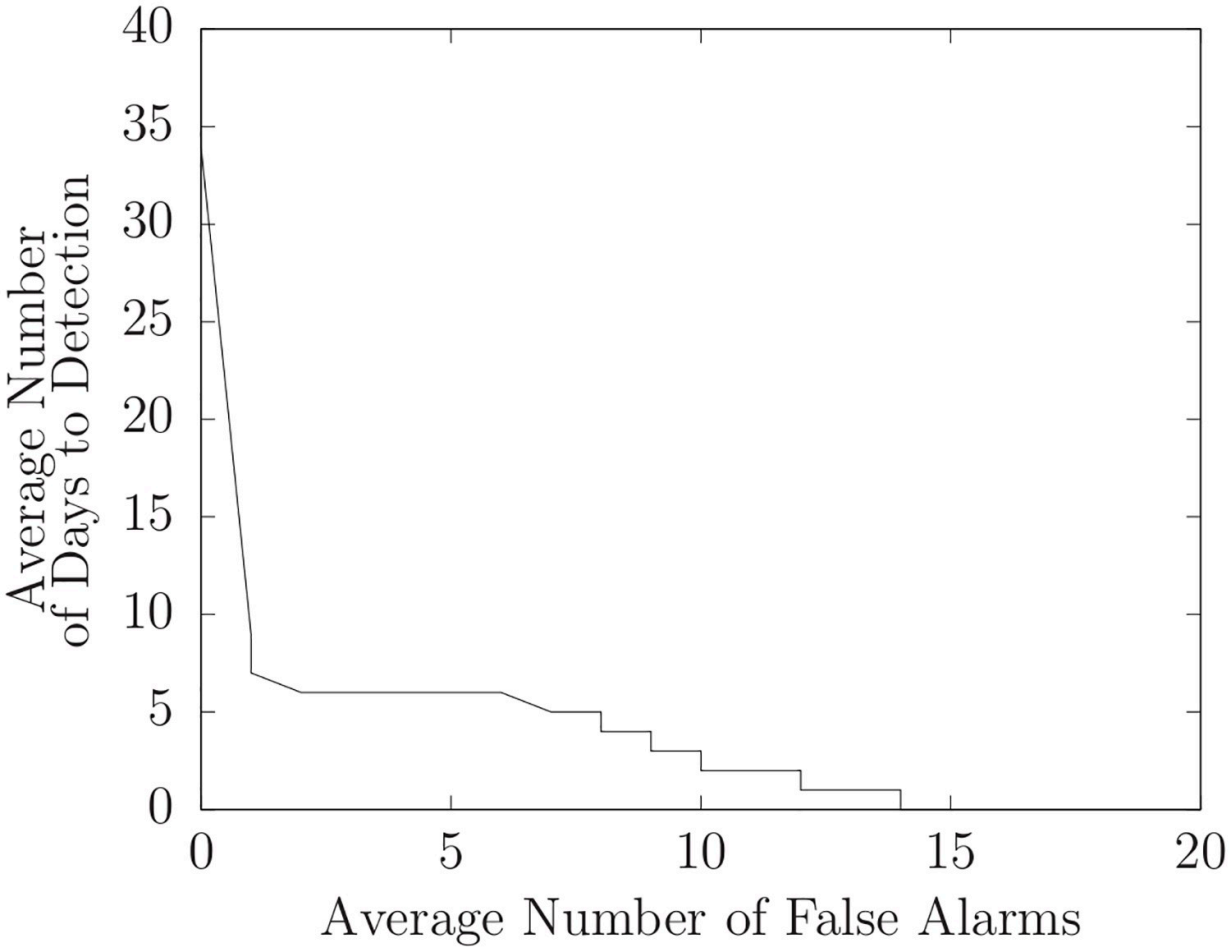
Average number of days to detection versus average number of false alarms for assumed unmodeled diseases.

We then ran DUDE on each of these fifteen datasets with various threshold values. For each threshold, we found the number of false alarms and the number of days to detection for each of the fifteen outbreaks. Then, for each threshold, we averaged the number of false alarms and the number of days to detection over the set of fifteen outbreaks. Thus, each threshold value produced a single point on the AMOC curve shown in Fig 5.

### Heuristic case selection

The AMOC curve in Fig 5 depends on assumptions we used to calculate the start dates of outbreaks. Nonetheless, such curves (when generated using identical assumptions) can be used to compare different techniques. In this section, we use AMOC curves to compare the basic algorithm described above to the basic algorithm enhanced with a heuristic.

An outbreak of an unmodeled disease is signaled by the presence of atypical patients in the monitor window. Because this is done by statistically comparing the features of the patients in the monitor window to the features of the patients in the baseline window, the presence of only a few atypical patients in the monitor window might not cause an alarm. This suggests that we can increase the effectiveness of DUDE by concentrating atypical patients in the monitor window and using them for detection. We have developed a heuristic method to do this. First, we compute the probability of each finding using all of the patients in the monitor window. Then we select patients in the monitor window whose findings are least likely given those probabilities. These selected patient cases comprise the new monitor window. Then, for comparison, we do the same with the patients in the baseline window to create a new baseline window comprised of the least likely patients.

**Fig 6:**
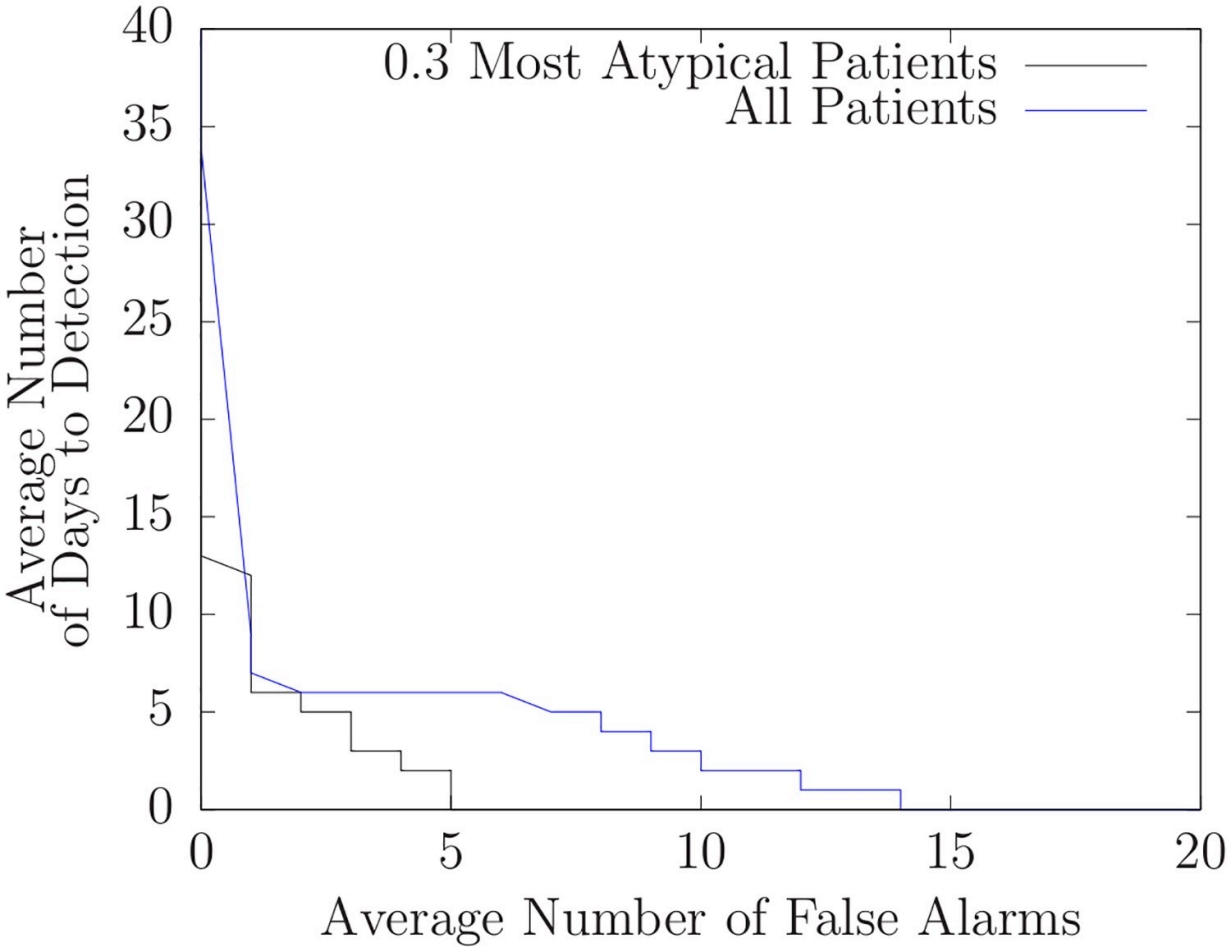
Average number of days to detection versus average number of false alarms for assumed unmodeled diseases with heuristic case selection using only the 30% most atypical patient records.

We applied this approach to the assumed unmodeled outbreaks for the five years of data by selecting the 30% most atypical patients. Fig 6 compares doing so to using all patients. Note that the AMOC graph corresponding to the 30% heuristically selected patients is lower at nearly every point than the AMOC curve without heuristic patient selection. This means that given thresholds for each approach that led to a particular number of false alarms, the heuristic approach will almost always have fewer days to detection. (We also applied this heuristic using the 10%, 50%, 70%, and 90% most atypical patients and obtained similar, but less, improvement). Although this heuristic worked well with the assumed unmodeled outbreaks (influenza, RSV, parainfluenza, and hMPV) for the five years of data we used, it failed to identify the unmodeled outbreak near day 106 of 2014-2015 that was identified by DUDE when it used all patient records. Although the likelihood of the presence of an unmodeled outbreak did increase slightly it never exceeded 1 indicating that the presence of an unmodeled disease was likely.

## Discussion

We conclude by noting three limitations of our approach and discuss how DUDE can be extended to overcome them.

Cases of a new, unmodeled disease are different from other cases that came before. The new disease might differ in an obvious way, such as displaying a rare symptom, or more subtly, such as in new combinations or rates of common symptoms. By design, DUDE identifies sets of cases with rates of symptoms that are statistically different from a baseline of earlier cases. However, it does this with a limited set of medical findings. While this highlights DUDE’s ability to identify outbreaks of diseases such as EV-D68 merely from common symptoms, it potentially misses diseases that are characterized by the presence of unusual symptoms (such as paralysis which is associated with EV-D68 [22]). To be more effective, DUDE will need access to a much wider range of symptoms than it currently uses. Of course, by using a large set of findings we risk overfitting, so this must be done in a probabilistically sound way.

Outbreaks of new (or old) diseases typically display an initial exponential rate of growth [14]. DUDE compares the *set* of cases in the monitor window with a baseline set of cases and generates an alarm if the presence of an unmodeled disease better explains the difference than simple variation. However, this ignores the daily increase in the number of cases of the unmodeled disease. It would be better if DUDE generated an alarm when it detects an *increasing number of cases of an unmodeled disease*. Doing so would increase the computational complexity since it will need to search over (the parameters of) a set of increasing curves and their start dates, but this can be ameliorated by heuristics that favor increasing disease curves that match increasing numbers of findings.

Cases of unmodeled diseases are often uncommon, at least initially. Because DUDE is built to identify new, unmodeled diseases it only considers records of patients who have definitively tested negative for all modeled diseases. However, since only a fraction of patients are comprehensively tested, this has the effect of ignoring much of the incoming data. A better approach would be to probabilistically model each of the known diseases and compute the daily expected number of each disease in the entire dataset. We could then compute the expected number of each finding—assuming that no unmodeled disease is present—then find the likelihood of an unmodeled disease based on how the actual distributions of findings differ from what would be expected if no unmodeled disease is present.

Finally, we note that we do not expect DUDE—or any system designed to detect an outbreak of an unmodeled disease—to work entirely autonomously. The tradoff between timeliness and false alarms dictates that occasional false alarms are inevitable and in times of heightened alertness (for instance, in the presence of anecdotal evidence) it may be prudent to lower the alarm threshold and examine the cases that generate alarms.

## Conclusions

We have demonstrated a Bayesian approach to modeling influenza-like illnesses, Detect Unmodeled Diseases from Evidence (DUDE), that is able to identify and characterize new, unmodeled diseases. We have measured its performance when detecting known diseases (while pretending to know nothing about them), and also shown that it is able (retroactively) to identify an outbreak of a new disease in a timely fashion. We have also identified future extensions that may improve its performance.

## Supporting information

S1 Appendix Derivation of Eqs 9 and 10. (PDF)
