# Supplementary material for: A Bayesian approach for detecting a disease that is not being modeled
Source: PLoS One. 2020 Feb 28;15(2):e0229658. doi: 10.1371/journal.pone.0229658 (PMC7048291; doi:10.1371/journal.pone.0229658)
Supplement: S1 Appendix — (PDF) [file pone.0229658.s001.pdf]

Derivations of Equations 9 and 10

To derive equation 9 note that:

$$P(\#f_m|\#\mathcal{B},\#\mathcal{U},\theta_{\mathcal{B},f}) = \int_0^1 \sum_{i=0}^{\#f_m} [bin(\#\mathcal{B},i,\theta_{\mathcal{B},f})\ bin(\#\mathcal{U},\#f_m-i,\theta_{\mathcal{U},f})]\ d\theta_{\mathcal{U},f} \quad (1)$$

$$= \sum_{i=0}^{\#f_m} [bin(\#\mathcal{B},i,\theta_{\mathcal{B},f}) \int_0^1 bin(\#\mathcal{U},\#f_m-i,\theta_{\mathcal{U},f})\ d\theta_{\mathcal{U},f}] \quad (2)$$

Happily, the integral reduces to a standard form with a simple solution (see, for instance, *Methods of Mathematics Applied to Calculus, Probability, and Statistics*, by Richard W. Hamming, Elsevier Academic Press, 1985):

$$\int_0^1 bin(\#\mathcal{U},\#f_m-i,\theta_{\mathcal{U},f})\ d\theta_{\mathcal{U},f} \quad (3)$$

$$= \int_0^1 C(\#\mathcal{U},\#f_m-i)\theta_{\mathcal{U},f}^{\#f_m-i}(1-\theta_{\mathcal{U},f})^{\#\mathcal{U}-(\#f_m-i)}\ d\theta_{\mathcal{U},f} \quad (4)$$

$$= C(\#\mathcal{U},\#f_m-i)\int_0^1 \theta_{\mathcal{U},f}^{\#f_m-i}(1-\theta_{\mathcal{U},f})^{\#\mathcal{U}-(\#f_m-i)}\ d\theta_{\mathcal{U},f} \quad (5)$$

$$= C(\#\mathcal{U},\#f_m-i)\frac{\Gamma(\#f_m-i+1)\Gamma(\#\mathcal{U}-(\#f_m-i)+1)}{\Gamma(\#f_m-i+1+\#\mathcal{U}-(\#f_m-i)+1)} \quad (6)$$

$$= C(\#\mathcal{U},\#f_m-i)\frac{\Gamma(\#f_m-i+1)\Gamma(\#\mathcal{U}-(\#f_m-i)+1)}{\Gamma(\#\mathcal{U}+2)} \quad (7)$$

$$= \frac{1}{\#\mathcal{U}+1} \quad (8)$$

Here, $C(a,b)$ is the number of combinations of a items taken b at a time, and $\Gamma(x)$ is Euler's gamma function which equals $(x-1)!$ for positive integers. Putting this together (and moving the constant out of the summation) gives

$$P(\#f_m|\#\mathcal{B},\#\mathcal{U},\theta_{\mathcal{B},f}) = \frac{1}{\#\mathcal{U}+1}\sum_{i=0}^{\#f_m} bin(\#\mathcal{B},i,\theta_{\mathcal{B},f}) \quad (9)$$

Equation 10 assumed that the counts of the findings, $\{\#f = c_f\}$, are conditionally independent. To show this, suppose there are findings $f_1,\ldots,f_n$. We want to show that:

$$P(\#f_1=c_1,\ldots,\#f_n=c_n|\#\mathcal{B},\#\mathcal{U}) = \prod_{i=1}^{n} P(\#f_i=c_i|\#\mathcal{B},\#\mathcal{U}) \quad (10)$$

We will show this by induction on n. It is trivial for $n=1$. Suppose it is true for $n-1$. We will suppress the conditions $\#\mathcal{B},\#\mathcal{U}$ which are common to all formulas, and assume they are part of the knowledge base.

We can express $P(\#f_1 = c_1, \ldots, \#f_n = c_n)$ as a sum of probabilities of truth-assignments to the $\{f_{i,j}\}$ that obey the constraints $\#f_1 = c_1, \ldots, \#f_n = c_n$. We can also separate each of these probabilities into a product of two terms where the left term is the probability of a truth-assignment to the $\{f_{1,j}\}$ and the right term is the probability of a truth-assignment to the $\{f_{i>1,j}\}$.

Let $\{\alpha_p\}$ be the set of all truth assignments to $\{f_{1,j}\}$ that satisfy the constraints, and let $\{\beta_q\}$ be the set of all truth assignments to $\{f_{i>1,j}\}$ that satisfy the constraints. Then:

$$P(\#f_1 = c_1, \ldots, \#f_n = c_n) = \sum_p \sum_q P(\alpha_p \cup \beta_q) \tag{11}$$

By the independence assumptions:

$$P(\#f_1 = c_1, \ldots, \#f_n = c_n) = \sum_p \sum_q P(\alpha_p) P(\beta_q) \tag{12}$$

Undistributing the $\{\alpha_p\}$:

$$P(\#f_1 = c_1, \ldots, \#f_n = c_n) = \sum_p [P(\alpha_p)[\sum_q P(\beta_q)]] \tag{13}$$

The inner sum is over the set of probabilities of truth assignments that satisfy $\#f_2 = c_1 \ldots, \#f_n = c_n$, so:

$$P(\#f_1 = c_1, \ldots, \#f_n = c_n) = \sum_p [P(\alpha_p)[P(\#f_2 = c_2 \ldots, \#f_n = c_n)]] \tag{14}$$

Undistibute again to get:

$$P(\#f_1 = c_1, \ldots, \#f_n = c_n) = [\sum_p P(\alpha_p)][P(\#f_2 = c_2 \ldots, \#f_n = c_n)] \tag{15}$$

Now, the sum is over the set of probabilities of truth assignments that satisfy $\#f_1 = c_1$, so:

$$P(\#f_1 = c_1, \ldots, \#f_n = c_n) = P(\#f_1 = c_1) P(\#f_2 = c_1 \ldots, \#f_n = c_n) \tag{16}$$

Finally, applying the inductive hypothesis gives:

$$P(\#f_1 = c_1, \ldots, \#f_n = c_n) = \prod_{i=1}^{n} P(\#f_i = c_i) \tag{17}$$
